# In Vitro Microscopical and Microbiological Assessment of the Sealing Ability of Calcium Silicate-Based Root Canal Sealers

**DOI:** 10.3390/jfb15110341

**Published:** 2024-11-12

**Authors:** Karin Christine Huth, Sabina Noreen Wuersching, Leander Benz, Stefan Kist, Maximilian Kollmuss

**Affiliations:** 1 Department of Conservative Dentistry and Periodontology, LMU University Hospital, LMU Munich, Goethestrasse 70, 80336 Munich, Germany; sabina.wuersching@med.uni-muenchen.de (S.N.W.); leander.benz@med.uni-muenchen.de (L.B.); maximilian.kollmuss@med.uni-muenchen.de (M.K.); 2 Department of Oral and Plastic Maxillofacial Surgery, Military Hospital Ulm, Academic Hospital of the University of Ulm, Oberer Eselsberg 40, 89081 Ulm, Germany; stefankist@bundeswehr.org

**Keywords:** sealers, bioceramic, biomaterials, endodontic materials, root canal filling, tricalcium silicates

## Abstract

This in vitro study evaluated the sealing ability and microleakage of calcium silicate-based sealers compared to an epoxy resin-based sealer. One hundred twenty-five roots from anterior teeth were chemo-mechanically prepared and divided into four groups: AH Plus (AH), ProRoot MTA (PR), Medcem MTA (MC), and Total Fill BC Sealer/BC-coated gutta-percha (TF); *n* = 30. Confocal laser scanning microscopy was used to measure sealer penetration at three horizontal levels in 10 roots per group, while glucose leakage over 30 days was assessed in 20 roots. A lateral compaction technique was used for most groups, except for TF, which employed a single-cone method. Data were analyzed using Python with a Kruskal–Wallis test and Dunn’s post hoc test. TF showed significantly greater penetration in the coronal and middle sections, while PR had the least penetration in the apical section. PR exhibited the highest canal circumference penetration, especially compared to MC and TF. Glucose leakage increased over time in all groups, with TF showing the highest permeability after 30 days. Overall, calcium silicate-based sealers PR, MC, and TF performed similarly to the epoxy resin standard AH, with all groups exhibiting decreasing penetration from coronal to apical and increased leakage over time.

## 1. Introduction

Microorganisms persisting in the root canal system are the main cause of failures of endodontic procedures [1,2]. Therefore, endodontic treatment involves rigorous disinfection and filling the root canals with gutta-percha and sealer to create a bacteria-tight root filling, preventing further colonization [2].

Sealers are highly flowable materials that help seal the root canal by filling gaps between gutta-percha cones and canal walls, evening out irregularities on canal walls, and closing lateral canals. They are crucial in preventing bacterial penetration and thus for the healing of the periapical region and the long-term success of root canal treatments [3,4]. Ideally, a sealer forms a hardened, thin, homogenous, and continuous layer, which tightens the region between the gutta-percha filling material and the canal wall deep into the adjacent dentinal tubules.

According to their chemical composition, sealers can be categorized into various material groups. Epoxy resin-based sealers constitute one category of low-viscosity root canal sealers and include widely employed materials such as AH Plus (Dentsply Sirona, York, PA, USA), which is considered the gold standard [5]. As advantages, their sealing properties and enhanced penetration depth into dentinal tubules, as well as dimensional stability, low solubility, and an antibacterial effect, have been reported [6,7,8]. However, as drawbacks, cytotoxic and pro-inflammatory effects have been noted, particularly during the initial setting stages [9,10]. Next to other sealer categories, hydraulic calcium silicate-based cements have gained popularity in endodontics for several indications to also be used as sealers. They are derived from mineral trioxide aggregate (MTA), a modified Portland cement composed of tricalcium silicate, dicalcium silicate, tricalcium aluminate, and other mineral oxides [11]. The dental market offers a wide range of different calcium silicate-based sealer compositions, including those with the incorporation of bioactive nanoparticles, such as Total Fill BC Sealer (BUSA, Savannah, GA, USA) and iRoot SP (Innovative BioCeramix Inc., Vancouver, BC, Canada). These sealers are notable for their high biocompatibility, a certain level of antimicrobial activity, signs of osteogenic and regenerative potential for certain brands, and effective sealing of the root canal system. This has been attributed to factors such as low solubility, alkaline pH, low film thickness, and high flow rate [8,12,13,14,15,16,17,18,19,20].

To reveal the quality of root canal fillings with respect to the completeness of the seal, different in vitro microscopic imaging techniques have been proposed, such as light microscopy, laser scanning microscopy, or scanning electron microscopy, as well as, recently, non-destructive high-resolution 3D (HR3D) imaging tools, such as µCT [21,22]. On the other side, microleakage models with different molecules are utilized to gauge the sealing effectiveness of root canal filling materials by quantifying the extent to which molecules can permeate the material. Among others, glucose is often used for such penetration models, as it is the main bacterial nutrient, and, therefore, the presence of glucose is crucial for possible bacterial reinfection. These models offer insights into the capacity of materials to prevent the infiltration of bacteria and their nutrients into the root canal space [23,24].

This study aims to assess the capability of the different calcium silicate-based sealers (CSBS) ProRoot MTA (PR) and Medcem MTA (MC), which were experimentally mixed to a sealer-suitable viscosity, and Total Fill BC Sealer (TF) to penetrate the dentinal tubules in comparison with the epoxy resin-based sealer AH Plus (AH). Two of the CSBS were experimentally mixed cements to achieve an appropriate sealer consistency. Specifically, the investigation entails evaluating the penetration depth into the dentinal tubules and the percentage of tubular penetration of the total root canal circumference visualized by fluorescence microscopy. Additionally, a glucose penetration model was employed to explore the resulting sealing efficacy. The null hypothesis was that the CSBS sealers perform equally as epoxy resin-based sealers.

## 2. Materials and Methods

### 2.1. Preparation of Tooth Specimens

A total of 125 freshly extracted, single-rooted human anterior teeth with similar size and root morphology from both the upper and lower jaws were gathered and preserved in Ringer’s solution supplemented with 0.2% sodium azide. Ethical clearance was obtained from the ethics committee of the Medical Faculty, Ludwig-Maximilians-University Munich (Registration No. 24-0763 KB). Prior to further use, radiographs of each tooth were taken to detect possible pathologies leading to exclusion from the study. Exclusion criteria were radicular resorptions, immature apices, fractures, caries of the root, or prior root canal treatments.

To achieve standardization of the teeth, the crowns of all teeth were removed using a diamond saw (LecoVari/Cut 50, LecoInstrumente, Mönchengladbach, Germany), leaving a consistent remaining root length of 15 mm. The working length was established at 14.5 mm, followed by the preparation of all root canals to a size of ISO 35.04 using MTwo rotary instruments (VDW, Munich, Germany). During preparation, the root canals were irrigated with 10 mL of 3% sodium hypochlorite (Aug. Hedinger, Stuttgart, Germany). A final rinse with 5 mL of 17% ethylenediaminetetraacetic acid (EDTA, Pharmacy of the University Hospital, Munich, Germany) was conducted to eliminate the smear layer.

### 2.2. Randomization, Experimental Groups, and Root Canal Filling

The collected teeth were randomly allocated to four equal-sized groups (*n* = 30), of which 10 teeth were used for investigating the penetration depth using a confocal laser scanning microscope (CLSM), while the remaining 20 teeth were designated for assessing glucose leakage. These group sizes were based on our previous findings on the microleakage of root-end fillings, where significant differences could be found for sample sizes of 15–20 teeth per group [24]. Additionally, five untreated teeth were employed as controls to ascertain the absence of autofluorescence during the analysis.

The root canal fillings were performed with gutta-percha and different sealer materials. The experimental groups are shown in Table 1. To utilize MTA-based materials as root canal sealers, achieving a lower viscosity compared to their conventional application is essential. To determine the optimal mixing ratio of MTA and distilled water to attain a consistency similar to AH and TF, a series of tests were conducted). The ideal mixing ratio was identified as 125 mg of cement powder combined with 80 µL of distilled water for PR and 78 µL of distilled water for MC. For each canal, an amount of 125 mg of cement was used, mixed, and inserted into the canal by a paper point to the full length of the canal and by feeding the central gutta-percha point all over.

The root canal fillings for the AH, PR, and MC groups were performed using the lateral compaction technique, employing stainless steel spreaders and standard gutta-percha points with an ISO size of 35.02 (VDW). In the case of TF, the manufacturer’s instructions were followed, and bioactive nanoparticle-coated gutta-percha points with an ISO size of 35.04 (Total Fill BC Points, American Dental Systems) were used with a single-cone technique.

For fluorescence visibility during laser scanning microscopy, 10 teeth of each group were filled with sealer containing 0.1% Rhodamine B (Merck, Darmstadt, Germany) before obturation. This was followed by X-ray images to verify the accurate length and homogeneity of the root canal filling in accordance with predetermined quality criteria [25] To allow the sealers to set, the teeth were placed in a humid chamber (60% humidity) at 37 °C for seven days.

**Table 1:** Class and composition of the test materials.

Experimental Group	Name and Manufacturer	Composition	Class
AH	AH Plus^®^ (Dentsply DeTrey GmbH, Konstanz, Germany)	Epoxide paste: Diepoxide, Calcium tungstate, Zirconium oxide, Aerosil, Pigment Amine paste: 1-adamantane amine, N,N′-dibenzyl-5-oxa-nonandiamine-1,9, TCD-Diamine, Calcium tungstate, Zirconium oxide, Aerosil, Silicone oil (manufacturer data sheet)	Epoxy-amin resin sealer (2-component material)
PR	ProRoot^®^ MTA (Dentsply DeTrey GmbH, Konstanz, Germany)	Bismuth oxide, tricalcium silicate, dicalcium silicate, calcium aluminate, calcium sulfate dyhydrated, trace elements (Fe, Ni, Cu, Sr) [26]	Calcium silicate-based sealer (2-component material)
MC	Medcem MTA (Medcem GmbH, Weinfelden, Switzerland)	Portland cement: tricalcium aluminate and silicate, dicalcium silicate, tetracalcium aluminoferrite, calcium oxide + zirconium oxide (radio opacifier) (manufacturer data sheet)	Calcium silicate-based sealer (2-component material)
TF	Total Fill^®^ BC Sealer^TM^/BC-coated gutta-percha (American Dental Systems, Vaterstetten, Germany)	Zirconium oxide, dicalcium and tricalcium silicates, calcium phosphate, calcium hydroxide, filler, thickening agents [17,27]	Bioceramic calcium silicate- based sealer (1-component material)

### 2.3. Solubility Measurements

Solubility measurements were performed according to DIN EN ISO 6876 as described before [24]. In brief, defined cement specimens were weighted and incubated at 37 °C in 50 mL double distilled water for 24 h. After evaporation of the discarded liquid in a pre-weighed Petri dish, the dish was weighed again, resulting in the weight of the components gone in solution, which was given in percent of the initial cement mass.

### 2.4. Fluorescence Microscopic Analysis

**Figure 1:**
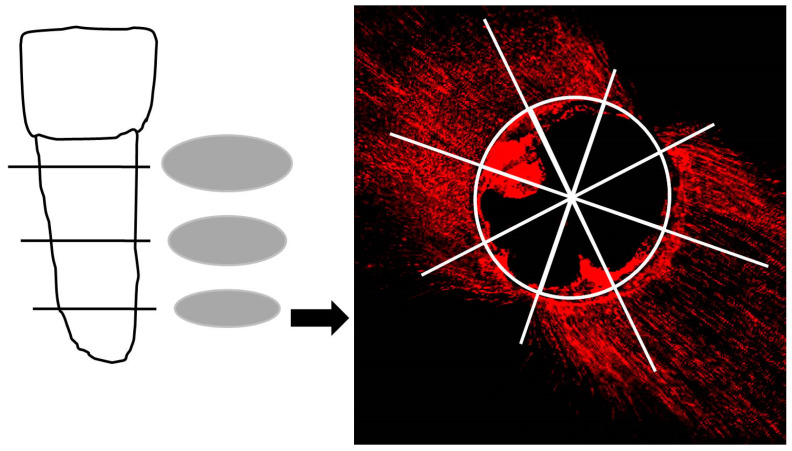
Each root underwent horizontal sectioning at three different distances from the apex: 4 mm, 8 mm, and 12 mm. An illustrative horizontal section obtained through CLSM is shown on the right. A total of 8 measurements were performed for each section, spaced at 45° intervals, as depicted in the accompanying pie chart.

For each group, 10 root samples were used to prepare microscopic sections (Figure 1). Using a diamond saw (LecoVari/Cut 50, LecoInstrumente) with continuous water cooling, three thin horizontal discs were obtained from each tooth. These discs were taken at distances of 4 mm, 8 mm, and 12 mm from the apex. The discs were affixed to microscope slides using TechnoVit 4000 (Heraeus Kulzer, Hanau, Germany). The coronal aspect of each section was polished with sandpaper discs (designated as 320, 600, and 1200 CAMI Grit) using the Leco VP 100 (LecoInstrumente) while ensuring continuous water cooling. After being left to air-dry for one week, the samples were subjected to analysis using a CLSM (LSM 510, Carl Zeiss AG, Jena, Germany).

When examining the coronal side of the sections, the penetration depth of the four different sealers, marked by the fluorescent dye Rhodamine B, was visualized with a laser wavelength of 543 nm. Regarding the penetration depths, measurements were taken at 8 different points spaced at 45° intervals (Figure 1). Additionally, the root canal circumference where material penetration occurred was determined and computed as a percentage of the total root canal circumference. Adobe Photoshop CS4 (version 11.0.1, Adobe Systems Software Ireland Ltd., Dublin, Ireland) was used for analysis. A reference scale of the same magnification as the samples was photographed and imported into the software. This facilitated the measurement of penetration depths and canal circumferences in micrometers (µm) in the three sections of the root canal.

### 2.5. Glucose Penetration Assay for Leakage Analysis

The remaining 20 root samples were employed for leakage analysis using a glucose penetration model as previously described [24]. Preliminary tests confirmed that the utilized sealers did not react with glucose.

**Figure 2:**
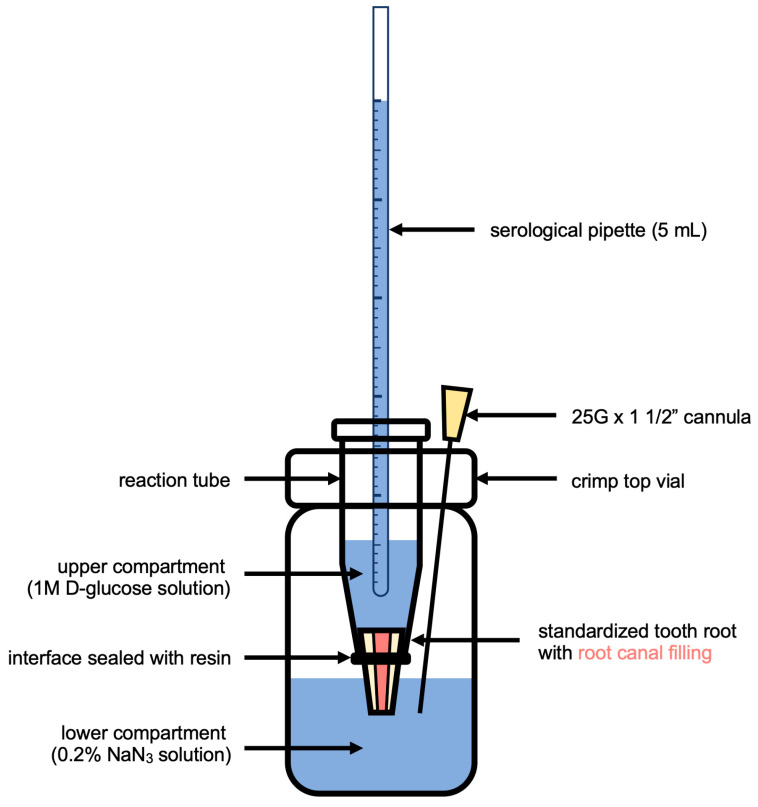
Diagram showing the experimental setup for assessing glucose microleakage in extracted human teeth with root canal treatment.

The coronal portion of each root was securely placed within an Eppendorf vial, which was trimmed at the apex and sealed with a temporary resin material (Luxatemp Automix plus, DMG, Hamburg, Germany). These Eppendorf vials, housing the roots, were then positioned within sterile crimp top vials containing 3.5 mL of H_2_O with 0.2% NaN_3_. The roots were arranged in a manner that the root tip came into contact with the aqueous solution. Serological pipettes were inserted into holes in the Eppendorf vials and filled with 5 mL of a 1 M aqueous solution of D-glucose, resulting in a hydrostatic pressure of 1.5 kPa on the experimental setup, which is shown in Figure 2. The entire assembly was relocated and maintained at a temperature of 37 °C and humidity level of 60% for a designated observation period of 30 days.

At intervals of 1, 7, 15, 20, and 30 days, samples were extracted from the lower compartment of the setup, and an equal volume of fresh NaN_3_ solution was replenished. Glucose concentrations within the lower compartment were assessed using an enzyme-based glucose Assay Kit (Glucose (HK) Assay Kit, Merck). Following the enzymatic reaction, the absorbance of produced NADH/H^+^ at 340 nm was measured utilizing a photometer (Varioskan, Thermo Fisher Scientific, Waltham, MA, USA). In this assay, the NADH/H^+^ concentration corresponds to the glucose concentration within the sample. All samples were measured in duplicate, and average values were computed from a standard curve with known glucose concentrations.

### 2.6. Statistical Analysis

Statistical analyses were performed in Python 3.8.8 using the packages *scipy*, *scikit*, *matplotlib*, and *seaborn* [28]. Data were tested for normal distribution with the Shapiro–Wilk test, and homogeneity of variances was assessed with Levene’s test. Multiple group comparisons were evaluated with the Kruskal–Wallis test, followed by Dunn’s post hoc test with a Bonferroni correction. For comparing microleakage between the groups, histograms with a binwidth of 0.05 mg/mL were computed to represent the distribution of the glucose concentration in the lower compartment for each day of measurement. The alpha level was set to 0.05 for all evaluations.

## 3. Results

Prior to the fluorescence microscopic image measurements, several aspects concerning the reliability of the method were tested: No autofluorescence was detected in the 5 control roots. All root canal fillings met the established criteria for correct length and homogeneity. The solubility measurements showed that AH and MC showed the least dissolved mass (<0.1%), followed by PR (0.5%), and TF showed the most (2.48%).

**Figure 3:**
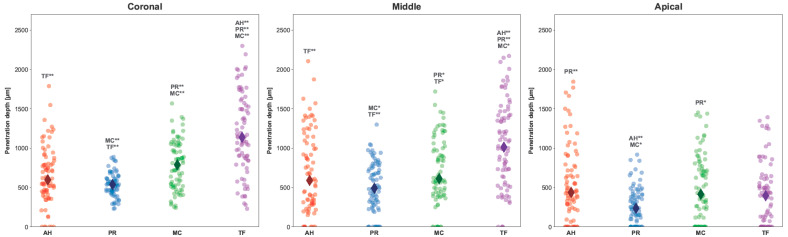
Strip plot presenting the penetration depths (µm) of each sealer into the dentinal tubules of the apical, middle, and coronal sections of the root canals. *p*-values obtained with Kruskal–Wallis test and Dunn’s post hoc test with a Bonferroni correction. *, *p* < 0.05; **, *p* < 0.001.

Sealer penetration depths into the dentinal tubules for the coronal, middle, and apical sections are shown in Figure 3. In the coronal and middle sections of the roots, TF showed significantly higher penetration depths compared to AH, PR, and MC. In all three sections, the lowest average penetration depth was in the PR group (*p* < 0.001 compared to MC and TF). In the apical section, PR showed the lowest average penetration depth (*p* < 0.001 compared to AH, and *p* < 0.05 compared to MC). The penetration depths of all other materials were in a similar range as AH. In general, all sealers showed a decrease in penetration depth from coronal to apical.

**Figure 4:**
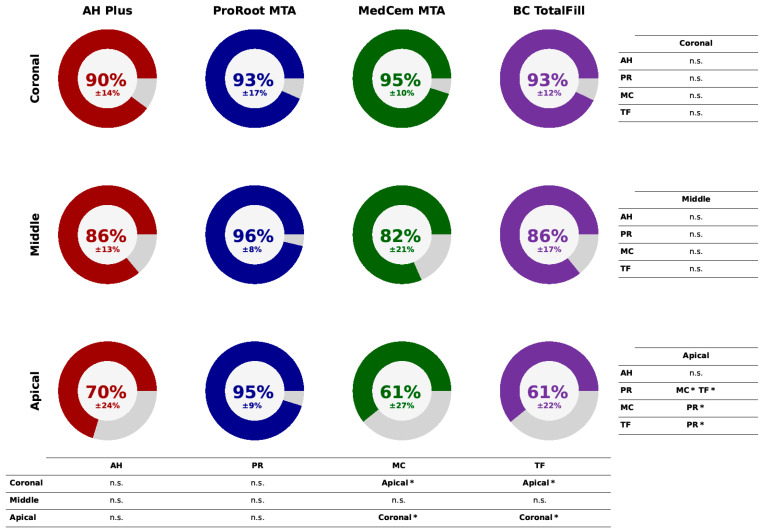
Average percentages of the circumference penetrated by the sealers in the apical, middle, and coronal regions of the root canals. Data shown as means and standard deviation. *p*-values obtained with Kruskal–Wallis test and Dunn’s post hoc test with a Bonferroni correction. *, *p* < 0.05; n.s., no significant differences.

The percentages of the root canal circumference where penetration occurred are displayed in Figure 4. In the apical section, PR showed a significantly higher circumference of penetration than MC and TF (*p* < 0.05), while no other significant differences were revealed in the middle or coronal sections between the sealer groups. However, within the MC and TF groups, the penetrated circumferences appeared to be significantly larger in the coronal than in the apical sections. The absolute data for the penetration depths and the penetrated circumferences in the coronal, middle, and apical sections are presented as Supplementary Materials.

**Figure 5:**
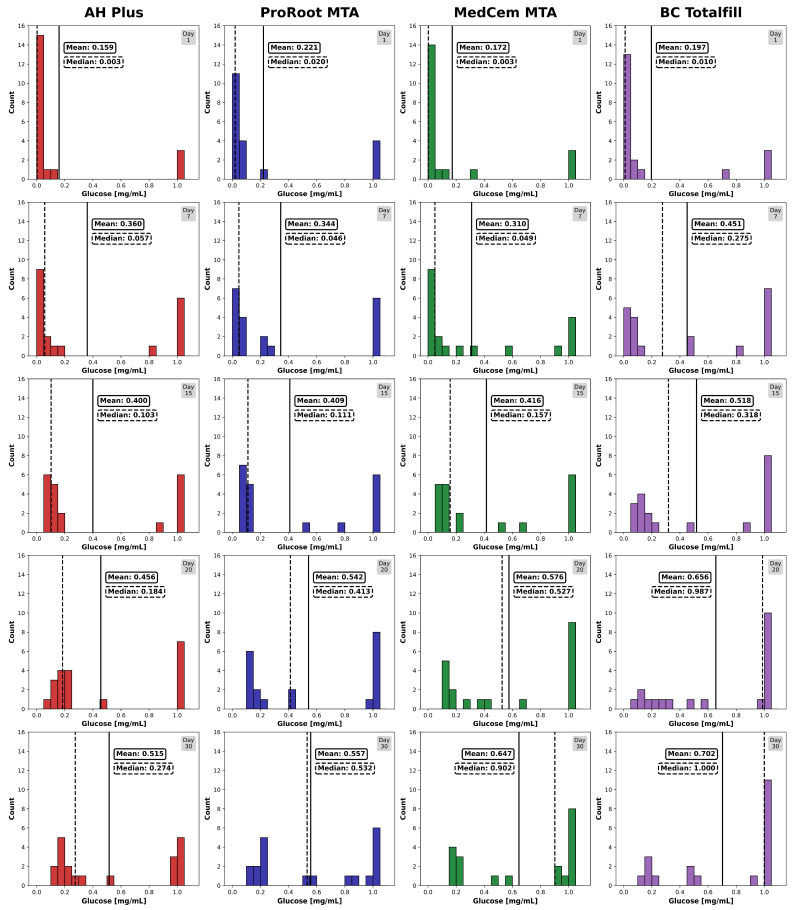
Glucose microleakage of root canal-treated teeth. Histograms represent the distribution of the glucose concentration (mg/mL) in the lower compartment for each day of measurement (*n* = 20 per group, binwidth = 0.05 mg/mL). The results for the different sealer groups are given from left to right. The change in glucose concentration over time (days 1, 7, 15, 20, and 30) is given from top to bottom for each group. The columns represent the number of specimens showing the specific glucose concentration. The vertical solid and plotted lines (black) give the mean and median concentration of penetrated glucose for each sealer group and time point.

The histograms showing the amount of glucose leaked through the root canal fillings indicate an increase in leaky specimens in all groups over the observation time of 30 days (Figure 5). TF showed the highest number of specimens with high glucose concentrations (1 mg/mL) in the lower compartment after 30 days, whereas AH, PR, and MC showed similar performances. Overall, the null hypothesis could be accepted.

## 4. Discussion

Our experiments showed that TF achieved significantly higher penetration depths in the coronal and middle root sections compared to the other sealers, while no differences in penetrated circumferences were observed among the sealer groups. In the apical third of the roots, TF had similar penetration depths as AH and MC, whereas PR had significantly the least. However, PR showed the highest percentage of penetrated canal circumference apically. Overall, the penetration depths decreased from coronal to apical in all groups. This pattern has been previously noted for epoxy resin, CSBS, and silicone-based sealers and can be explained anatomically by a decrease in tubule density and diameter towards the apex, increased sclerosis, and major canal branches [29], as well as less effective smear layer removal, which hinders tubule penetration [30]. The penetration depths of AH in our study were comparable to recent studies, also showing a decrease from coronal to apical. For example, mean values were reported as 864.14 µm (coronal), 669.08 µm (middle), and 400.52 µm (apical) [31]. Our finding that TF exhibited superior penetration depths aligns with several other studies, which used different brands of CSBS like Bio-C-Sealer or iRoot SP [32,33,34]. In contrast, some studies reported AH as having superior penetration depths compared to CSBS like MTA-plus and Bioroot RCS [35] or found no difference compared to epoxy resin-based sealers [36,37]. Despite these conflicting results, the deeper penetration of TF and, to a lesser extent, MC in our study can be attributed to the hydrophilicity, higher flow rate, and alkaline nature of CSBS, which degrade tubular collagen [17], compared to the epoxy resin-based sealers. For instance, iRoot SP—another brand name for TF—has been reported to meet ISO 6876:2012 requirements with a flow rate of more than 17 mm and a film thickness lower than 50 µm, though this was thicker than that reported for AH [38]. A recent study revealed a mean flowability as high as 26.28 mm for TF and 24.33 mm for AH, along with a film thickness of 25.00 µm for TF and 26.33 µm for AH [8]. Additionally, the study showed that TF maintained a more alkaline pH than AH over the experimental period of 3 months.

While current research [39] predominantly uses 0.1% Rhodamine B as a fluorescence dye for microscopic image analysis, including all aforementioned studies, doubts have been raised regarding its suitability for sealer staining in CLSM measurements [40]. They reported that the dye could leach out of the sealers (in this case, AH, TF, and BioRoot RCS) deeper into the tubules, thereby overestimating the penetration depths and creating the illusion of the root canal sealers greatly interweaving with the root dentin. In this context, our findings from the preliminary solubility measurements may be of interest, with TF dissolving more mass (2.46%) than the others (AH, MC < 0.1%, and PR 0.5%). TF solubility is thus only slightly below the ISO 6876 requirements of less than 3% weight loss for sealers after immersion in water (24 h). We cannot exclude the possibility that some dye dissolved along with the mass, leading to the aforementioned uncertainty. However, since the same method was applied to all tested sealers, the comparability between the sealers and the validity of the observed differences should be maintained. The flow characteristics of the sealers, which are crucial for achieving sealing—alongside other physical and chemical parameters like surface tension, particle size, and setting time—were also described not to be affected by a 0.1% Rhodamine B dye [41].

To evaluate the completeness of the sealers in penetrating the canal wall, we found that PR covered a higher percentage of canal wall circumference with penetrated tubules in the apical sections compared to MC and TF. All sealers were administered in the same amount, with AH, MC, and PR groups using lateral compaction for root canal filling and the TF group using a single cone technique with a bioactive nanoparticle-coated gutta-percha point according to manufacturer instructions. In the MC and TF groups, the percentage of penetrated circumference was higher in the coronal sections than in the apical sections, which corresponds with the general finding that penetration depths decrease from coronal to apical.

As the aim of the sealers and solid obturation material is to create a bacteria-tight seal of the root canal system, we proceeded with a glucose penetration test, which we previously used to evaluate the sealing effectiveness of calcium silicate-based cements as root-end filling materials [24]. This method is advantageous because it is non-destructive and glucose permeation represents the main nutrient source for microorganisms [23]. Our results showed an increase in leaky specimens in all groups, including the CSBS groups, over the 30-day observation period, which has been reported before [3]. In our experiments, TF exhibited significantly higher permeability than AH and PR, with no significant difference compared to MC. However, no significant differences were found between AH, PR, and MC. The higher solubility of TF, as determined by our own measurements with a 2.46% dissolving mass, only marginally meets the ISO requirements mentioned above. This solubility has been reported to be even higher in other studies, consistent with the generally higher solubility of CSBS compared to epoxy resin-based sealers [17]. However, we could not confirm this for PR and MC, which showed distinctly lower solubility than TF in our study, as previously reported [42]. This higher solubility of TF may be related to our finding of distinctly increased glucose permeability compared to AH, PR, and MC, as reported before [43,44]. It might also be attributed to higher porosity or slight volume changes [42].

Despite the limitations of the penetration depth and circumference measurements, which did not account for factors such as voids, gaps, the 3D spatial distribution of material along the canal wall down to the apex, and water-induced expansion, the glucose penetration test might have mitigated these drawbacks to some extent. This is because it measures exactly what we aim to avoid: the permeability of the root filling to glucose, which is a necessary nutrient for bacteria. In general, all in vitro methods for the determination of sealing ability are only models and cannot predict the clinical outcome: Microscopic methods, as well as penetration methods with different substances, are not able to give a definitive estimation of the tightness of a filled root canal. It is mandatory to conduct different experiments to obtain an estimation of a possible clinical effect.

The methods used included cross-sectioning of the treated teeth, which likely resulted in specimen damage and partial loss of material. Today, non-destructive high-resolution 3D imaging (HR3D), such as that facilitated by µCT, is a method for displaying root canals for volumetric analyses [21,22,45,46]. Nonetheless, results have been reported to be variable and difficult to compare regarding sealer type or filling technique [45]. The even more recent phase-contrast-enhanced synchrotron radiation µCT (PCE µCT), despite its costs and technical demands, holds promise for more detailed endodontic research [47,48,49]. Another limitation of our study is the shortness of the observation period of only 30 days. Root fillings in a clinical setting have to stay bacteria-tight for many years to allow the long-term success of the treatment. In this light, in vitro data as presented by us cannot be transferred directly to a clinical setting, but they allow an insight into the performance of these sealer materials to conduct further investigations in this field. Another factor that may limit clinical transfer is that MC and PR were experimentally mixed to a sealer-suitable viscosity. However, given their successful use as cements in root repair, applying them as sealers throughout the canal also seems reasonable [8,24].

## 5. Conclusions

In light of the mentioned study limitations, we can conclude from our results that the CSBS PR and MC, in their experimentally determined mixing ratios, and the premixed TF performed similarly to the epoxy resin-based gold-standard sealer AH. In general, the penetration depths decreased from coronal to apical, and all specimens showed an increase in glucose leakage over time. The use of CSBS may, therefore, be a clinical alternative to conventional sealers, especially in situations dealing with root damages or similar conditions.

## Data Availability

The original contributions presented in the study are included in the article/supplementary material, and further inquiries can be directed to the corresponding author.

## Supplementary Materials

The following supporting information can be downloaded at: https://www.mdpi.com/article/10.3390/jfb15110341/s1, Table S1: Penetration depths of sealers into dentinal tubules, circumference of the root canals, and circumference penetrated by the sealers in the coronal, middle, and apical regions of the root canals. Data are shown as means and standard deviation as well as median.

## References

[1] Nair P.N.R. (2006). On the causes of persistent apical periodontitis: A review. Int. Endod. J..

[2] Gulabivala K., Ng Y.L. (2023). Factors that affect the outcomes of root canal treatment and retreatment-A reframing of the principles. Int. Endod. J..

[3] Khayat A., Lee S.J., Torabinejad M. (1993). Human saliva penetration of coronally unsealed obturated root canals. J. Endod..

[4] Barborka B.J., Woodmansey K.F., Glickman G.N., Schneiderman E., He J. (2017). Long-term Clinical Outcome of Teeth Obturated with Resilon. J. Endod..

[5] Viapiana R., Moinzadeh A.T., Camilleri L., Wesselink P.R., Tanomaru Filho M., Camilleri J. (2016). Porosity and sealing ability of root fillings with gutta-percha and BioRoot RCS or AH Plus sealers. Evaluation by three ex vivo methods. Int. Endod. J..

[6] Santos J.N., Tjäderhane L., Ferraz C.C., Zaia A., Alves M., De Goes M., Carrilho M. (2010). Long-term sealing ability of resin-based root canal fillings: Sealing ability of endodontic fillings. Int. Endod. J..

[7] Prestegaard H., Portenier I., Ørstavik D., Kayaoglu G., Haapasalo M., Endal U. (2014). Antibacterial activity of various root canal sealers and root-end filling materials in dentin blocks infected ex vivo with Enterococcus faecalis. Acta Odontol. Scand..

[8] Wuersching S.N., Diegritz C., Hickel R., Huth K.C., Kollmuss M. (2022). A comprehensive in vitro comparison of the biological and physicochemical properties of bioactive root canal sealers. Clin. Oral Investig..

[9] Camargo C.H.R., Oliveira T.R., Silva G.O., Rabelo S.B., Valera M.C., Cavalcanti B.N. (2014). Setting Time Affects In Vitro Biological Properties of Root Canal Sealers. J. Endod..

[10] Konjhodzic-Prcic A., Gorduysus O., Kucukkaya S., Atila B., Muftuoglu S., Zeybek D. (2015). In Vitro Comparison of Cytotoxicity of Four Root Canal Sealers on Human Gingival Fibroblasts. Med. Arh..

[11] Chaudhari P.S., Chandak M.G., Jaiswal A.A., Mankar N.P., Paul P. (2022). A Breakthrough in the Era of Calcium Silicate-Based Cements: A Critical Review. Cureus.

[12] Zhang H., Shen Y., Ruse N.D., Haapasalo M. (2009). Antibacterial Activity of Endodontic Sealers by Modified Direct Contact Test Against Enterococcus faecalis. J. Endod..

[13] Zhang W., Li Z., Peng B. (2010). Ex vivo cytotoxicity of a new calcium silicate-based canal filling material: Cytotoxicity of iRoot SP. Int. Endod. J..

[14] Assmann E., Böttcher D.E., Hoppe C.B., Grecca F.S., Kopper P.M.P. (2015). Evaluation of Bone Tissue Response to a Sealer Containing Mineral Trioxide Aggregate. J. Endod..

[15] McMichael G.E., Primus C.M., Opperman L.A. (2016). Dentinal Tubule Penetration of Tricalcium Silicate Sealers. J. Endod..

[16] Mestieri L.B., Gomes-Cornélio A.L., Rodrigues E.M., Salles L.P., Bosso-Martelo R., Guerreiro-Tanomaru J.M., Tanomaru-Filho M. (2015). Biocompatibility and bioactivity of calcium silicate-based endodontic sealers in human dental pulp cells. J. Appl. Oral Sci..

[17] Donnermeyer D., Bürklein S., Dammaschke T., Schäfer E. (2019). Endodontic sealers based on calcium silicates: A systematic review. Odontology.

[18] Donnermeyer D., Schemkämper P., Bürklein S., Schäfer E. (2022). Short and Long-Term Solubility, Alkalizing Effect, and Thermal Persistence of Premixed Calcium Silicate-Based Sealers: AH Plus Bioceramic Sealer vs. Total Fill BC Sealer. Materials.

[19] Urban K., Neuhaus J., Donnermeyer D., Schäfer E., Dammaschke T. (2018). Solubility and pH Value of 3 Different Root Canal Sealers: A Long-term Investigation. J. Endod..

[20] Bose R., Ioannidis K., Foschi F., Bakhsh A., Kelly R.D., Deb S., Mannocci F., Niazi S.A. (2020). Antimicrobial Effectiveness of Calcium Silicate Sealers against a Nutrient-Stressed Multispecies Biofilm. J. Clin. Med..

[21] De-Deus G., Santos G.O., Monteiro I.Z., Cavalcante D.M., Simões-Carvalho M., Belladonna F.G., Silva E.J.N.L., Souza E.M., Licha R., Zogheib C. (2022). Micro-CT assessment of gap-containing areas along the gutta- percha-sealer interface in oval-shaped canals. Int. Endod. J..

[22] De-Deus G., Souza E.M., Silva E.J.N.L., Belladonna F.G., Simões-Carvalho M., Cavalcante D.M., Versiani M.A. (2022). A critical analysis of research methods and experimental models to study root canal fillings. Int. Endod. J..

[23] Xu Q., Fan M.W., Fan B., Cheung G.S., Hu H.L. (2005). A new quantitative method using glucose for analysis of endodontic leakage. Oral Surg. Oral Med. Oral Pathol. Oral Radiol. Endod..

[24] Kollmuss M., Preis C.E., Kist S., Hickel R., Huth K.C. (2017). Differences in physical characteristics and sealing ability of three tricalcium silicate-based cements used as root-end-filling materials. Am. J. Dent..

[25] Unal G.C., Kececi A.D., Kaya B.U., Tac A.G. (2011). Quality of root canal fillings performed by undergraduate dental students. Eur. J. Dent..

[26] Belío-Reyes I.A., Bucio L., Cruz-Chavez E. (2009). Phase composition of ProRoot mineral trioxide aggregate by X-ray powder diffraction. J. Endod..

[27] Yap W.Y., Che Ab Aziz Z.A., Azami N.H., Al-Haddad A.Y., Khan A.A. (2017). An in vitro Comparison of Bond Strength of Different Sealers/Obturation Systems to Root Dentin Using the Push-Out Test at 2 Weeks and 3 Months after Obturation. Med. Princ. Pract..

[28] Van Rossum G., Drake F.L. (2009). PYTHON 3 Reference Manual.

[29] Mjör I.A., Smith M.R., Ferrari M., Mannocci F. (2001). The structure of dentine in the apical region of human teeth. Int. Endod. J..

[30] Tuncel B., Nagas E., Cehreli Z., Uyanik O., Vallittu P., Lassila L. (2015). Effect of endodontic chelating solutions on the bond strength of endodontic sealers. Braz. Oral Res..

[31] Verma A., Arora A., Taneja S. (2024). Comparative evaluation of dentinal tubule penetration and push-out bond strength of new injectable hydraulic calcium disilicate based root canal sealer: A single blinded in vitro study. J. Oral Biol. Craniofacial Res..

[32] Akcay M., Arslan H., Durmus N., Mese M., Capar I.D. (2016). Dentinal tubule penetration of AH Plus, iRoot SP, MTA fillapex, and guttaflow bioseal root canal sealers after different final irrigation procedures: A confocal microscopic study. Laser Surg. Med..

[33] El Hachem R., Khalil I., Le Brun G., Pellen F., Le Jeune B., Daou M., El Osta N., Naaman A., Abboud M. (2019). Dentinal tubule penetration of AH Plus, BC Sealer and a novel tricalcium silicate sealer: A confocal laser scanning microscopy study. Clin. Oral Investig..

[34] Caceres C., Larrain M.R., Monsalve M., Pena Bengoa F. (2021). Dentinal tubule penetration and adaptation of bio-C sealer and AH-plus: A Comparative SEM evaluation. Eur. Endod. J..

[35] Arikatla S.K., Chalasani U., Mandava J., Yelisela R.K. (2018). Interfacial adaptation and penetration depth of bioceramic endodontic sealers. J. Conserv. Dent..

[36] Kim H., Kim E., Lee S.J., Shin S.J. (2015). Comparisons of the retreatment efficacy of calcium silicate and epoxy resin-based sealers and residual sealer in dentinal tubules. J. Endod..

[37] Chen H., Zhao X., Qiu Y., Xu D., Cui L., Wu B. (2017). The tubular penetration depth and adaption of four sealers: A scanning electron microscopic study. BioMed Res. Int..

[38] Zhou H.M., Shen Y., Zheng W., Li L., Zheng Y.F., Haapasalo M. (2013). Physical properties of 5 root canal sealers. J. Endod..

[39] Awati A.S., Dhaded N.S., Mokal S., Doddwad P.K. (2024). Analysis of the depth of penetration of an epoxy resin-based sealer following a final rinse of irrigants and use of activation systems: An in vitro study. J. Conserv. Dent. Endod..

[40] Donnermeyer D., Schmidt S., Rohrbach A., Berlandi J., Bürklein S., Schäfer E. (2021). Debunking the Concept of Dentinal Tubule Penetration of Endodontic Sealers: Sealer Staining with Rhodamine B Fluorescent Dye Is an Inadequate Method. Materials.

[41] Weller R.N., Tay K.C., Garrett L.V. (2008). Microscopic appearance and apical seal of root canals filled with gutta-percha and ProRoot Endo Sealer after immersion in a phosphate-containing fluid. Int. Endod. J..

[42] Zamparini F., Siboni F., Prati C., Taddei P., Gandolfi M.G. (2019). Properties of calcium silicate-monobasic calcium phosphate materials for endodontics containing tantalum pentoxide and zirconium oxide. Clin. Oral Investig..

[43] Deniz Sungur D., Purali N., Cosgun E., Calt S. (2016). Push-out bond strength and dentinal tubule penetration of different root canal sealers used with coated core materials. Restor. Dent. Endod..

[44] Yanpiset K., Banomyong D., Chotvorrarak K., Srisatjaluk R.L. (2018). Bacterial leakage and micro-computed tomography evalua- tion in round-shaped canals obturated with bioceramic cone and sealer using matched single cone technique. Restor. Dent. Endod..

[45] Keleş A., Keskin C. (2020). Presence of voids after warm vertical compaction and single-cone obturation in band-shaped isthmuses using micro-computed tomography: A phantom study. Microsc. Res. Tech..

[46] Holmes S., Gibson R., Butler J., Pacheco R., Askar M., Paurazas S. (2021). Volumetric Evaluation of 5 Root Canal Obturation Methods in TrueTooth 3-dimensional–Printed Tooth Replicas Using Nano–computed Tomography. J. Endod..

[47] Moinzadeh A.T., Farack L., Wilde F., Shemesh H., Zaslansky P. (2016). Synchrotron-based Phase Contrast-enhanced Micro–Computed Tomography Reveals Delaminations and Material Tearing in Water-expandable Root Fillings Ex Vivo. J. Endod..

[48] Moinzadeh A.T., Zerbst W., Boutsioukis C., Shemesh H., Zaslansky P. (2015). Porosity distribution in root canals filled with gutta percha and calcium silicate cement. Dent. Mater..

[49] Soares A.P., Bitter K., Lagrange A., Rack A., Shemesh H., Zaslansky P. (2020). Gaps at the interface between dentine and self-adhesive resin cement in post-endodontic restorations quantified in 3D by phase contrast-enhanced micro-CT. Int. Endod. J..

