# Supplementary material for: In Vitro Microscopical and Microbiological Assessment of the Sealing Ability of Calcium Silicate-Based Root Canal Sealers
Source: J Funct Biomater. 2024 Nov 12;15(11):341. doi: 10.3390/jfb15110341 (PMC11595059; doi:10.3390/jfb15110341)
Supplement: Supplementary file 1 [file jfb-15-00341-s001.zip › jfb-3242851-supplementary.pdf]

Supplementary Material:

Table S1:

Penetration depths of sealers into dentinal tubules, circumference of the root canals and circumference penetrated by the sealers in the coronal, middle and apical region of the root canals. Data shown as means and standard deviation as well as median.

Sealer	Region	Penetration depth (mean ± SD, median, µm)	Circumference root canal (mean ± SD, µm)	Penetrated Circumference (mean ± SD, µm)
**AH**	**Coronal**	646.63 ± 362.14<br>598	4424.50 ± 933.19	4007.39 ± 1162.17
	**Middle**	695.46 ± 510.69<br>590.5	2945.87 ± 722.23	2570.32 ± 834.18
	**Apical**	561.56 ±471.46<br>438	2427.65 ± 460.60	1722.24 ± 731.89
**PR**	**Coronal**	537.08 ±152.78<br>537.5	4437.24 ± 679.23	4135.07 ± 986.14
	**Middle**	505.84 ± 317<br>490.5	2602.80 ± 553.61	2522.50 ± 620.19
	**Apical**	262.31 ±235.53<br>235.5	2151.69 ± 505.88	2052.63 ± 540.25
**MC**	**Coronal**	783.36 ±310.13<br>789.5	3657.20 ± 881.74	3449.70 ± 845.02
	**Middle**	703.83 ± 422.95<br>612.5	2475.32 ± 309.98	2019.77 ± 586.90
	**Apical**	489.54 ± 430.03<br>414.5	2322.24 ± 560.54	1389.42 ± 593.07
**TF**	**Coronal**	1182.11 ± 502.26<br>1138.5	3304.15 ± 631.21	3075.78 ± 727.79
	**Middle**	1042.89 ± 545.26<br>1013.5	2681.76 ± 436.30	2306.06 ± 601.23
	**Apical**	399.3 ± 393.58<br>396.5	1677.10 ± 301.73	1025.37 ± 427.48
